# 阵发性睡眠性血红蛋白尿症补体抑制治疗研究进展

**DOI:** 10.3760/cma.j.cn121090-20240903-00332

**Published:** 2025-01

**Authors:** 忆萌 史, 凤奎 张

**Affiliations:** 1 国家血液系统疾病临床医学研究中心，血液与健康全国重点实验室，细胞生态海河实验室，中国医学科学院血液病医院（中国医学科学院血液学研究所），天津 300020 National Clinical Research Center for Blood Diseases, State Key Laboratory of Experimental Hematology, Haihe Laboratory of Cell Ecosystem, Institute of Hematology & Blood Diseases Hospital, Chinese Academy of Medical Sciences & Peking Union Medical College, Tianjin 300020, China; 2 天津医学健康研究院，天津 301600 Tianjin Institutes of Health Science, Tianjin 301600, China

## Abstract

阵发性睡眠性血红蛋白尿症（PNH）为一获得性克隆性造血异常，以往大部分PNH患者主要接受支持性治疗，生活质量差，死亡率高。下游补体C5抑制剂的出现则极大地改变了PNH的自然病程，在改善临床症状的同时，明显降低严重并发症的发生，提高了患者的生存率。尽管C5抑制剂在控制血管内溶血（IVH）和提高血红蛋白（HGB）水平方面效果显著，但是上游补体成分C3介导的血管外溶血（EVH）使得近半数治疗患者仍需要输血。上游补体抑制剂在控制IVH的同时，减少了C3片段的沉积，从而解决EVH的问题，进一步改善了PNH患者的临床结局。但是，由于补体激活的级联反应以及上游补体抑制剂治疗后PNH红细胞的比例累积增加，可能会增加发生更为严重的突破性溶血事件的风险。此外，补体抑制剂可能会增加感染风险，这也是当前需要关注的问题。

## Abstract (en)

Paroxysmal nocturnal hemoglobinuria（PNH）is an acquired clonal hematologic disorder. Historically, most PNH patients mainly received supportive treatment, which led to poor quality of life and high mortality rates. The advent of downstream complement C5 inhibitors has dramatically changed the natural course of PNH. These inhibitors alleviate clinical symptoms, significantly reduce severe complications, and improve survival rates. Despite their ability to control intravascular hemolysis and enhance hemoglobin levels, approximately half of the treated patients still require blood transfusions due to extravascular hemolysis（EVH）mediated by upstream complement component C3. Upstream complement inhibitors not only control IVH but also reduce the deposition of C3 fragments, thereby solving the problem of EVH and further improving the clinical outcomes of PNH patients. However, due to the cascade of complement activation and the increased accumulation of PNH red blood cells after treatment with upstream complement inhibitors, there may be an increased risk of more severe breakthrough hemolysis events. Additionally, the potential risk of infections associated with complement inhibitors is another issue that needs to be addressed.

阵发性睡眠性血红蛋白尿症（paroxysmal nocturnal hemoglobinuria，PNH）为一获得性克隆性造血异常，以慢性持续性血管内溶血阵发性加剧、经常伴发骨髓衰竭和高风险血栓形成为特征[1]–[2]。造血干细胞PIG-A基因突变引起糖基磷脂酰肌醇（glycosylphosphatidylinositol，GPI）锚蛋白合成缺陷，致使红细胞表面GPI锚定蛋白（GPI-anchored proteins，GPI-AP），包括补体调节蛋白CD55和CD59缺失，最终导致补体介导的红细胞溶解和高血栓形成倾向[2]–[4]。异基因造血干细胞移植是唯一可以治愈PNH的方法，但其治疗风险高，仅适合小部分患者，因而以往大部分PNH患者主要接受支持性治疗，生活质量差，死亡率高，中位生存期为10～15年[5]–[6]。补体抑制剂的出现极大地改变了PNH的自然病程，在改善临床症状的同时，明显降低严重并发症的发生，将患者的生存率提高到与普通人群相似的水平[7]。本文聚焦近年补体抑制剂治疗PNH的研究进展，介绍不同补体抑制剂的疗效及安全性如下。

一、下游补体抑制剂

下游补体抑制剂通过阻断C5的激活，阻止末端膜攻击复合物（membrane attack complex, MAC）的形成。对接受C5抑制剂治疗的509例PNH患者随访20年的数据显示，C5抑制剂可降低PNH患者血栓事件的发生率，将血栓栓塞发生率从治疗前的7.37/100患者年降低至0.73/100患者年，并且能够显著降低PNH患者的死亡率，当除外存在需要治疗的骨髓衰竭患者后，PNH患者的生存率与性别和年龄匹配的对照组相似，10年累积生存率为95.85％，19年累积生存率为96.24％[8]。

依库珠单抗（Eculizumab）是首个被批准用于治疗PNH的C5单克隆抗体，治疗1周即可明显降低乳酸脱氢酶（lactate dehydrogenase，LDH）水平，迅速控制血管内溶血（intravascular hemolysis，IVH），显著减少输血需求，降低85％的血栓栓塞事件发生率，改善患者的生活质量，将生存率提高到与同年龄同性别健康人群相似的水平[7],[9]–[11]。对PNH患者将近15年的随访数据显示，依库珠单抗可使患者的生存率提高49％，并可降低约60％的血栓栓塞事件/主要不良血管事件发生风险[12]。但在依库珠单抗治疗期间，仅有51％的患者可以脱离红细胞输注，20％～30％的患者可实现HGB正常化[8]–[10],[13]。11％～29％的患者可出现与C5抑制效果欠佳相关的突破性溶血（break through hemolysis，BTH），包括药代动力学BTH（药物低谷浓度）和药效学BTH（感染、炎症等引起补体激活增强并突破C5阻断）[14]–[15]，可能需要缩短给药间隔或是提高维持剂量以维持终末补体抑制[16]。此外，依库珠单抗对携带C5多态性的患者无效以及2周1次静脉维持用药的不便捷也是该治疗策略存在的问题[17]。

雷夫利珠单抗（Ravulizumab）是长效的人源化C5单克隆抗体，作用靶点与依库珠单抗相同，半衰期延长至49.7 d，维持治疗静脉给药间隔时间延长至8周[18]，极大地方便了患者，目前已逐渐成为替代依库珠单抗的一线选择。两项非劣效性临床研究显示，在初治（301研究）[19]及既往接受依库珠单抗治疗（302研究）[20]的患者中，在所有关键疗效终点上，雷夫利珠单抗均不劣于依库珠单抗，且安全性相当。此外，雷夫利珠单抗可完全抑制游离C5，通过消除游离C5相关的BTH来降低BTH的总体发生风险[21]。雷夫利珠单抗皮下注射制剂则进一步优化了给药方式，初步临床研究显示，雷夫利珠单抗皮下注射制剂在药代动力学上不劣于静脉制剂，PNH患者可从静脉注射依库珠单抗或雷夫利珠单抗转换为皮下注射雷夫利珠单抗而不会失去疗效[22]。

新型C5抑制剂可伐利单抗（Crovalimab）采用SMART-Ig再循环技术，使其能够与C5高效结合，促进内皮细胞的摄取从而有效清除C5，而可伐利单抗在新生儿Fc受体介导下得以回收，使其半衰期延长至58.7 d，每4周1次皮下注射维持给药即可快速、完全、持续地抑制C5，提高了治疗的便利性，达到血药浓度稳定的患者可实现自行给药[23]–[24]。COMMODORE研究显示，在初治及既往接受补体抑制剂治疗的PNH患者中，在主要疗效终点，即控制溶血和脱离输血方面，可伐利单抗均不劣于依库珠单抗[25]–[27]。基于COMMODORE 2、3研究结果，可伐利单抗在中国获批，用于治疗初治的溶血性PNH。不同于依库珠单抗和雷夫利珠单抗，可伐利单抗与C5 β链上的表位结合，因此对具有C5多态性的患者依旧有效，但需要注意的是，由于结合表位不同，在从依库珠单抗转换为可伐利单抗时，可能会出现药物-靶点-药物复合物（drug-target-drug complexes，DTDC）反应[24],[27]。

帕泽利单抗（Pozelimab）/特度鲁单抗（Tesidolumab）/Coversin的结合位点不同于依库珠单抗，因此可能成为因C5多态性而对依库珠单抗无效的PNH患者的有效治疗药物，初步研究证实了其在携带C5基因多态性患者中的有效性[28]–[30]。新型C5抑制剂的安全性及有效性尚需进一步临床研究验证。

Cemdisiran是一种皮下注射的小干扰RNA（siRNA），可沉默C5 mRNA，抑制肝细胞合成补体成分C5，将血清C5水平减少90％以上[31]。Ⅰ/Ⅱ期临床研究发现，Cemdisiran单药治疗虽然能够降低LDH，但不能完全控制IVH，探索性研究结果显示，Cemdisiran和依库珠单抗联合给药可以减少依库珠单抗的用药剂量，将给药间隔从每2周1次延长至每4周1次，并且可以改善对依库珠单抗反应不佳的PNH患者的治疗反应[31]–[32]，推测Cemdisiran与C5单抗联合使用可能对PNH患者更有益。目前，Cemdisiran和帕泽利单抗的联合用药方案已在两项Ⅱ期临床研究中显示出初步疗效。在从帕泽利单抗单药治疗过渡到联合用药的PNH患者中，有95％（21/22）的患者IVH得到控制，90％（20/22）患者的符合HGB稳定标准（未接受红细胞输注，HGB降低不超过20 g/L）[33]。而在既往接受依库珠单抗治疗的PNH患者中，Cemdisiran和帕泽利单抗联合用药可使所有患者（5/5）的IVH得到控制，达到HGB稳定，且没有报道BTH，包括既往接受较高剂量依库珠单抗治疗的2例患者（每2周1200 mg和1500 mg）[34]。Cemdisiran和C5单抗联合用药方案的有效性及安全性需进一步评估。

尽管下游补体抑制剂能够阻断末端MAC的形成，但不影响补体替代途径（alternative pathway，AP）的早期反应，包括C3的活化、裂解及沉积，过多的C3片段可在PNH红细胞表面沉积，随后通过C3的调理作用导致红细胞在肝脾网状内皮系统中被吞噬破坏，最终引起血管外溶血（EVH）[13]。因此，在接受C5抑制剂治疗的PNH患者中，仅有少数患者HGB正常化，大部分患者的红细胞寿命明显缩短，网织红细胞计数（ARC）和胆红素水平持续升高[35]–[36]。

二、上游补体抑制剂

末端补体抑制剂无法解决C3介导的EVH，很大程度上推动了靶向补体上游级联反应的药物研发。上游补体抑制剂在抑制MAC形成的同时，减少了C3片段的沉积，从而解决EVH的问题，进一步改善PNH患者的临床结局。

（一）C3抑制剂

C3抑制剂Pegcetacoplan能够与C3及其激活片段C3b结合，抑制C3的裂解和补体激活下游效应物的生成，从而控制C3b介导的EVH和终末补体介导的IVH[37]。Ⅰ期和Ⅱ期临床研究显示，Pegcetacoplan能够有效控制初治以及接受依库珠单抗治疗PNH患者的IVH和EVH，在治疗患者中可以观察到PNH红细胞表面C3片段沉积减少[38]–[39]。PEGASUS研究结果表明，在依库珠单抗治疗反应欠佳的PNH患者中，Pegcetacoplan单药治疗在16周时显著改善HGB（调整后最小二乘均值差38.4 g/L，*P*<0.001），脱离输血的患者比例更高（85％对15％），更多患者的HGB、ARC、LDH和总胆红素水平恢复至正常，并且有更多患者的疲劳症状得到了改善[40]。在随后32周的开放标签期中，Pegcetacoplan单药治疗改善HGB及溶血标志物的疗效得以维持，患者从依库珠单抗转换为Pegcetacoplan治疗后，其HGB、ARC、FACIT疲劳评分均得到明显改善[41]。PRINCE研究比较了Pegcetacoplan与支持治疗（如输血、糖皮质激素和补充造血原料等）在初治PNH患者中的疗效及安全性，结果显示，在26周时，Pegcetacoplan治疗组有更多患者达到了HGB稳定，脱离红细胞输注，LDH、ARC、FACIT疲劳评分以及生活质量评分改善均优于支持治疗组[42]。根据对PEGASUS和PRINCE研究的事后分析显示，Pegcetacoplan能够使更多初治以及依库珠单抗治疗患者的D-二聚体正常化，降低血栓事件的发生率[43]，但目前尚缺乏Pegcetacoplan在降低血栓形成风险方面的疗效数据。相较于静脉补体抑制剂，Pegcetacoplan改进了给药途径，但大剂量、频繁的皮下注射给药（推荐1 080 mg每周2次）仍是需要注意的问题，研究显示Pegcetacoplan治疗期间最常见的不良事件是注射部位反应（37％）[40]。

（二）B因子抑制剂

伊普可泮（Iptacopan）为口服给药的B因子抑制剂，临床研究显示，伊普可泮单药治疗或是作为C5抑制剂的附加疗法均能很好的控制初治及C5抑制剂反应欠佳的PNH患者的IVH和EVH。

在既往接受C5抑制剂治疗但反应欠佳的患者中，伊普可泮附加治疗在13周时能显著降低LDH，改善HGB，80％（8/10）患者HGB恢复正常。在随后的扩展研究期间，血液学反应得以维持，包括7例停用依库珠单抗而接受伊普可泮单药治疗的患者[44]。APPLY-PNH研究结果显示，第24周时，伊普可泮单药治疗（200 mg，每日2次）在主要疗效终点方面均优于C5抑制剂，HGB较基线升高≥20 g/L和HGB≥120 g/L的患者比例更高。且与抗C5治疗相比，伊普可泮单药治疗组有更多患者脱离输血，更好地提高HGB、降低ARC和改善FACIT疲劳评分[45]。对APPLY-PNH研究进行血液学反应类别分析，结果显示，71％（44/62）接受伊普可泮单药治疗的患者获得完全血液学反应，C5抑制剂治疗患者均未获得完全血液学反应，54.3％患者仅为部分血液学反应[46]。在之后24周延长期内，伊普可泮治疗组血液学及临床获益得以维持，从C5抑制剂转换为伊普可泮治疗患者也获得了与伊普可泮单药治疗组相当的血液学及临床症状改善[47]。基于Ⅲ期APPLY-PNH试验，FDA批准伊普可泮作为首个口服单药疗法，用于治疗成年PNH患者，包括既往接受过C5抑制剂治疗的患者。

对于初治患者，伊普可泮单药治疗可有效降低患者的LDH，所有可评估疗效的12例患者均达到了主要疗效终点，第12周时LDH水平较基线降低≥60％，同时改善患者的HGB和溶血标志物水平[48]。后续12个月中期结果显示，伊普可泮降低LDH的疗效可维持至少52周[49]。APPOINT-PNH研究中，在24周时，92％（31/33）的患者达到主要终点，HGB较基线升高至少20 g/L，63％（19/33）患者达到次要终点，HGB上升至≥120 g/L。其他疗效终点也进一步支持伊普可泮单药治疗的有效性，包括98％的患者脱离输血，HGB升高43 g/L，FACIT疲劳评分改善10.8分，LDH较基线变化-83.6％[45]。62.5％（25/40）患者获得完全血液学反应[46]。基于APPOINT-PNH全球及中国亚组的研究结果，伊普可泮在中国获批用于治疗初治的成年PNH患者。

（三）D因子抑制剂

Danicopan是首创的口服D因子抑制剂，在治疗初治PNH的Ⅱ期临床研究中，Danicopan单药治疗可以显著降低LDH［基线：5.7×正常范围上限（ULN），d 28：1.8×ULN，d 84：2.2×ULN］，改善贫血（基线：98 g/L，d 28：109 g/L，d 84：141 g/L）。可能由于其药代动力学及药效学特性，Danicopan单独应用无法完全控制IVH[50]，推测可以利用C5抑制剂控制IVH，同时加用Danicopan解决EVH。Ⅱ期临床研究结果显示，在第24周，Danicopan附加治疗明显提高了对依库珠单抗反应不佳患者的HGB，减少了血制品输注需求，胆红素、ARC及临床症状均得到改善[51]。Ⅲ期ALPHA研究评估了在依库珠单抗或ravulizumab基础上加用口服Danicopan的疗效，在第12周时，相较于安慰剂组，Danicopan附加治疗显著提高了HGB，HGB升高≥20 g/L以及脱离输血的患者比例更高，溶血标志物及临床症状均得到明显改善[52]。在随后为期12周的治疗期2内，安慰剂组患者转而接受Danicopan治疗，在第24周，继续接受Danicopan治疗组的血液学获益维持不变，安慰剂-Danicopan组HGB得到明显改善，35％的患者HGB增加≥20 g/L，90％的患者脱离输血，输血需求明显减少[53]。

与Danicopan相比，第二代D因子抑制剂Vemircopan与D因子的结合亲和力更高，抑制作用更强。Ⅱ期临床研究的初步结果显示，Vemircopan单药治疗可以有效控制初治PNH患者的IVH，将LDH降低至几乎正常水平，同时抑制EVH，改善HGB，降低ARC[54]。BCX9930也是一种口服小分子D因子抑制剂，在初治PNH患者中，单药治疗可以明显改善HGB，降低LDH，改善疲劳评分和ARC。对于C5抑制剂治疗欠佳的患者，BCX9930的加用可以明显减少红细胞输注，提高HGB，且在停用C5抑制剂后，血液学反应仍可维持，提示BCX9930单药治疗PNH的可能性[55]。

上游补体抑制剂抑制了缺陷红细胞的破坏导致PNH红细胞的比例累积增加，可能会增加发生更为严重的BTH事件的风险。此外，如果末端补体成分抑制不完全，单个活化C5的逸出仅导致单个MAC的形成，而由于补体激活的级联反应，近端补体成分的逃逸可能会催化多个下游补体成分的活化，最终形成多个MAC，从而引发严重的BTH事件。

PEGASUS研究报道，在16周时，41例Pegcetacoplan组患者有4例发生BTH，其中3例因溶血停药；在随后32周的开放标签期，77例患者有15例发生BTH，其中3例停药[40]。与在C5抑制剂治疗患者中观察到的BTH不同，Pegcetacoplan组中观察到的BTH更为严重，LDH高达ULN的10～15倍，更接近于未治疗的PNH患者中发生的严重IVH[56]。对PEGASUS研究中BTH事件进一步分析提示，基线时较高的疾病活动度可能会增加Pegcetacoplan治疗患者的溶血风险，超说明书的依库珠单抗用量、可检测到的CH50以及在过去1年内输血次数≥4次，可能可以作为溶血事件的预测指标[57]。最近的小样本研究显示，对于治疗期间发生急性BTH的患者，Pegcetacoplan 1 080 mg单次静脉注射或者1 080 mg连续3 d皮下注射的强化疗法可能是潜在的解决方案[58]。

伊普可泮的BTH发生频率以及严重程度似乎低于Pegcetacoplan，APPLY-PNH长期数据显示，在48周的伊普可泮治疗期间，7％（7/96）的患者发生BTH，所有患者均未停用药物[47]。APPOINT-PNH研究未报道BTH的发生[45]。而PEGASUS报道在Pegcetacoplan 48周的治疗期内，24％（19/80）的患者发生BTH，其中6例患者停药[41]。鉴于PNH患者补体抑制治疗的终生性，并且补体扩增事件随时可能发生，需长期监测随访以了解上游补体抑制剂发生BTH的总体风险以及严重程度。

三、其他补体抑制剂

近端补体抑制剂存在严重BTH的发生风险，联合抑制补体级联反应的上、下游可能可以解决单一补体抑制相关的安全问题。KP104是一种双功能补体抑制剂，由人源化的C5单克隆抗体和补体负性调节因子H的结构域组成，有效抑制补体末端途径（terminal pathway, TP）及AP的激活。Ⅱ期临床研究评估了KP104在初治PNH患者中的疗效，中期结果表明，到第16～17周，所有15例患者的HGB较基线明显上升，均脱离输血，60％（9/15）的患者HGB≥120 g/L，所有患者的LDH水平均下降至接近正常（低于1.5×ULN）。在随后的扩展治疗阶段，18例患者完成了24/26周的最优生物剂量治疗，HGB明显提升（较基线升高66 g/L），89％的患者HGB恢复正常，所有患者的LDH水平始终维持在接近正常水平。在给药一周内ARC迅速下降至正常水平（低于ULN），并在整个治疗期内得以维持，提示KP104对EVH的有效预防。在安全性方面，治疗期间没有出现3级及以上治疗相关不良事件，有2例BTH事件发生，考虑与胃肠炎及共存的骨髓增殖性肿瘤相关。没有报道严重感染事件，整体耐受性良好[59]。

甘露糖结合凝集素相关丝氨酸蛋白酶-3（MASP-3）将D因子前体转化为成熟的补体D因子，从而激活AP。与其他补体成分不同，MASP-3不是急性期反应物，其浓度在炎症环境中不会改变，理论上可以避免急性期出现的突破性病例。OMS906是高选择性的MASP-3单克隆抗体，Ⅰb期临床研究中期分析结果显示，对于初治PNH患者，OMS906治疗4周即可明显改善HGB，降低LDH。延长治疗至24周，血液学效应得以维持，HGB持续上升，11例患者中8例HGB恢复至正常水平。没有BTH及严重感染事件的报道[60]。

四、补体抑制剂的感染风险

补体抑制剂阻断了补体激活级联反应，可能增加感染风险，尤其是含荚膜的奈瑟菌如脑膜炎奈瑟菌和淋病奈瑟菌，因此在开始补体抑制剂治疗前至少2周应接种脑膜炎奈瑟菌疫苗以降低感染风险。需要注意的是，部分接种疫苗的患者仍发生了脑膜炎奈瑟菌感染，因此有学者建议在补体抑制剂治疗期间可预防性使用抗生素[61]。

对依库珠单抗治疗患者随访8年的数据显示，79例患者中2例发生了脑膜炎奈瑟菌感染，脑膜炎奈瑟菌感染发生率为0.9/100患者年，因此建议PNH患者在治疗期间常规进行预防性抗生素治疗[7]。依库珠单抗治疗PNH的10年药物警戒分析显示，依库珠单抗治疗患者脑膜炎奈瑟菌的感染发生率为0.24/100患者年，致死性脑膜炎奈瑟菌的感染发生率为0.03/100患者年，与健康人群相比，接受依库珠单抗治疗患者感染脑膜炎奈瑟菌的风险可高出1000～2000倍。另外，淋病奈瑟菌（1.7％）以及其他奈瑟菌的感染（1.1％）也有报道[62]。

Ⅰb/Ⅱ期临床研究显示，39例患者中2例在雷夫利珠单抗治疗期间发生了脑膜炎奈瑟菌感染，而Ⅲ期301、302研究及其2年的随访研究均未报道脑膜炎奈瑟菌感染病例[19]–[20],[63]–[64]。目前，临床试验未报告可伐利单抗治疗期间脑膜炎奈瑟菌感染病例。在应用C5抑制剂的3130个治疗年中，10例患者共发生了11例脑膜炎奈瑟菌的感染，其中1例死亡，脑膜炎奈瑟菌的感染发生率为0.35/100患者年[8]。

相较于C5抑制剂，近端补体C3抑制剂会对三种补体活化途径均有影响，可能会增加患者感染风险。目前PEGASUS和PRINCE研究均未报道Pegcetacoplan脑膜炎奈瑟菌感染病例，总体感染发生率与依库珠单抗差异无统计学意义[40]。B、D因子抑制剂选择性地靶向AP，能够保留经典途径和凝集素途径的先天免疫功能，可能会降低感染风险，B、D因子抑制剂相关的临床研究尚未报道荚膜细菌感染的病例。目前关于近端补体抑制剂感染风险的数据有限，尚需真实世界的长期随访监测数据以充分评估其感染风险。

五、总结与展望

目前，PNH的补体抑制治疗研究逐渐从静脉制剂的末端补体抑制剂转向口服制剂的上游补体抑制剂，虽然能够更好地控制IVH和预防EVH，提高患者用药的便利性，但是可能会增加发生严重血管内BTH的风险。由于C5分子与MAC形成之间呈线性关系，如果存在近端补体抑制不完全，同时靶向AP和TP是减少严重血管内BTH发生风险的理想选择。另外，目前的上游补体抑制剂半衰期较短，其血浆水平可仅因一次错过剂量就降低至抑制阈值以下，一次突破即可迅速发生大规模的溶血事件，延长上游补体抑制剂的半衰期，减慢药物清除速度以及时监测并处理发生的突破性事件，可能可以避免出现严重的BTH。

尽管补体抑制剂的应用能够明显改善PNH患者的临床症状，提高生存率，但其潜在的BTH和感染风险、疾病本身伴随的骨髓衰竭等问题仍是未来需要注意的问题。对末端补体持续而完全的抑制是PNH治疗的关键，提高补体抑制剂在体内的靶向性和生物利用度以优化抑制效果，减少突破性事件，将有助于进一步改善PNH患者的临床结局。
